# Antihyperglycemic and Antioxidative Stress Effects of *Erythrophleum africanum* (Fabaceae) Trunk Bark Powder Fractions on High‐Calorie Diet‐Induced Type 2 Diabetes in Rats

**DOI:** 10.1002/fsn3.70290

**Published:** 2025-05-14

**Authors:** Mathieu Sini, Faustin Dongmo, Clemence Mvongo, Michel Archange Fokam Tagne, Pierre Jidibe, Angèle Foyet Fondjo, Paul Aimé Noubissi, René Kamgang

**Affiliations:** ^1^ Department of Biological Sciences, Faculty of Science University of Ngaoundere Ngaoundere Cameroon; ^2^ Department of Life Sciences Higher Teacher Training College, University of Bertoua Bertoua Cameroon; ^3^ Department of Applied Sciences for Health Higher Institute of Applied Sciences, University Institute of Gulf of Guinea Douala Cameroon; ^4^ Department of Zoology and Animal Physiology, Faculty of Science University of Buea Buea Cameroon; ^5^ Laboratory of Endocrinology and Radioisotopes Institute of Medical Research and Medicinal Plants Studies (IMPM) Yaounde Cameroon

**Keywords:** antihyperglycemic activity, antioxidant activity, *Erythrophleum africanum*, high sugar diet

## Abstract

Diabetes mellitus is a persistent and chronic metabolic disease characterized by high blood glucose levels. The aim of this work was to evaluate the antihyperglycemic and antioxidative stress effects of *Erythrophyllum africanum* trunk bark powder fractions in diabetes‐induced rats. 30 male rats subdivided into 6 groups of five rats each received daily a sweetened hypercaloric diet supplemented with sucrose (4 g/kg bw), except for the normal control, which received a normal diet. Normal and diabetic controls subsequently received distilled water (10 mL/kg bw *per os*), the positive control received metformin (20 mg/kg bw *per os*) and the test rats received powder fractions (≤ 50 μm ≤ 50–120 μm) or unsieved powder of *E. africanum* (300 mg/kg bw *per os*) for 7 weeks. Dexamethasone (0.2 mg/kg) was administered intraperitoneally once a week from the third week, except for the normal control, which received saline. Fasting blood glucose, lipid profile, and biochemical parameters of oxidative stress were assessed during and at the end of treatment. Blood glucose levels of the animals at the 7th week were 0.92 ± 0.03, 1.52 ± 0.08, 0.78 ± 0.04, 0.77 ± 0.03, 1.13 ± 0.03, and 0.40 ± 0.01 g/L in the normal control, diabetic control, metformin‐treated animals, ≤ 50 μm fraction, 50–120 μm fraction, and unsieved powder, respectively. Powder fraction ≤ 50 μm significantly improved (*p* < 0.01) the lipid profile (decrease in triglyceride and LDL cholesterol levels, an increase in HDL cholesterol levels) by reducing the atherogenic index. *E. africanum* has antihyperglycemic and antioxidative stress effects and would be less toxic to the liver and kidneys. The fine powder (≤ 50 μm) of *E. africanum* could be used as a food additive to prevent the occurrence of diabetes in vulnerable patients.

## Graphical abstract

The powder fraction of Erythrophleum africanum with a size less than 50 μm prevents the occurrence of type 2 diabetes induced by the combination of a high‐calorie diet and the intake of dexamethasone. *E. africanum* has antihyperglycemic and antioxidative stress effects and improves the lipid profile by reducing the atherogenic index. This plant would be less toxic to the liver and kidneys.

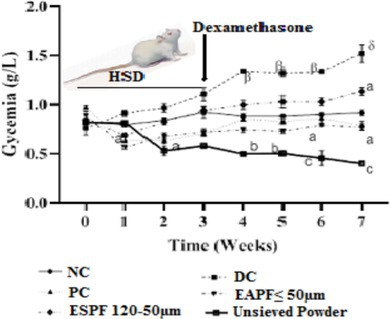

## 1 Introduction

Diabetes mellitus is a persistent and progressive metabolic disease characterized by high blood glucose levels due to a defect in insulin production or a defect in insulin action, or both (Almaghrabi et al. 2021). The classification of diabetes describes two major types (ADA 2010) including type 1 (T1D) and type 2 diabetes (T2D) which is the most common form (90% of cases). T2D is characterized by insulin resistance (Tenenbaum et al. 2018). Some drugs such as glucocorticoids may be implicated in the pathophysiology of type 2 diabetes. Dexamethasone is one of the glucocorticoids that induces hyperglycemia by increasing hepatic gluconeogenesis through increased expression and activity of phosphoenolpyruvate carboxylase and glucose‐6‐phosphatase and by reducing the sensitivity of insulin secretion (Clore and Thurby‐Hay 2009; Sari et al. 2021; Zhang and Karam 2021). Diabetes mellitus is one of the major global health emergencies of the 21st century, with a significant increase in cases and a mortality rate that is increasingly considerably high worldwide (Liu et al. 2021). According to the 10th International Diabetes Federation (IDF) report, diabetes mellitus caused 6.7 million deaths worldwide in 2021, with an estimated prevalence of 643 million people affected in 2030 and 783 million in 2045, if no effective management measures are taken (IDF 2021). According to the WHO Global Diabetes Report 2016, 24 million Africans are diagnosed with diabetes, and this number is expected to increase by 129% to 55 million by 2045 (OMS 2016). In the absence of treatment, permanent hyperglycemia can be the cause of the installation of oxidative stress by activation of the metabolic pathways of formation of reactive oxygen species (Delattre et al. 2001; Omar et al. 2023). Many drugs (hypoglycemic sulfonamides and biguanides) are available for the treatment of type 2 diabetes, but almost all have adverse side effects (Chen et al. 2003). Prevention of diabetes mellitus is possible by respecting hygiene and dietary rules (Foussier and Zergane 2020) and treatment in conventional medicine is based on the use of hypoglycemic agents such as insulin (Bello et al. 2011) and oral antidiabetics (biguanides, sulphonamides, glinides, thiazolidinediones, etc.) (Haq et al. 2021). Metformin is one of the first‐line treatments for type 2 diabetes due to its hypoglycemic effects, well‐established safety profile, and relatively low cost. Metformin inhibits hepatic gluconeogenesis in a substrate‐selective manner and alters cellular redox balance (Lamoia and Shulman 2021). However, this drug management, although offering the expected therapeutic effects, nevertheless has many side effects such as diarrhea, vomiting, heart failure, and the risk of severe hypoglycemia (Kancherla et al. 2017; Seaquist et al. 2013). Prolonged consumption of metformin can lead to anorexia, vomiting, gastrointestinal effects, and vitamin B12 malabsorption (Dhananjay et al. 2014). Therefore, there is an urgent need to identify natural resources and study their potential pharmacological properties in order to develop new, less toxic antidiabetic treatments. Many herbal preparations have been the subject of several experimental and preclinical trials in the treatment of type 2 diabetes (Kamgang et al. 2008; Mvongo et al. 2016; Ngakou Mukam et al. 2025). *Erythrophleum africanum* is a medium‐sized tree of the Fabaceae family found in much of tropical Africa, from Senegal east to Sudan, and south to Central Africa, Kenya, Tanzania, and Southern Africa (Maroyi 2019). *Erythrophleum africanum* is a pyrophyte species that only establishes in open environments, often after the passage of severe fires. *E. africanum* has pubescent rachises, petioles, and petiolules. The leaflets are non‐accumulate and measure about 6.5 × 3.5 cm. The blade is often pubescent on both sides with clearly visible secondary veins. The flowers are about 4 mm, yellowish‐white to reddish in color with free sepals. The pods measure 2.2–4.5 × 7–19 cm, have a median implantation of the stipe, and contain 3–5 seeds with a thin endosperm (Gorel et al. 2015). *E. africanum* is traditionally used in the treatment of several pathologies such as cardiovascular diseases, sexually transmitted diseases, various inflammations, diabetes, leprosy, goiter, dysentery, diarrhea, and as an astringent (Son 2019). The aim of the present study was therefore to evaluate the antihyperglycemic and antioxidative stress effects of the trunk bark powder fractions of *Erythrophleum africanum* on high‐calorie diet‐induced type 2 diabetes in rats. Secondary metabolites are generally more concentrated in the finer fraction of the plant, and therefore this fraction will have more activities (Jones and Kinghorn 2006).

## 2 Materials and Methods

### 2.1 Experimental Animals

The experimental animals consisted of male albino Wistar rats strain aged eight to 10 weeks and weighing between 140 and 190 g. These animals came from the animal house of the Faculty of Sciences of the University of Ngaoundere (Cameroon) and were raised under conditions of room temperature (25°C), humidity (50%–60%), sufficient ventilation, and a light/dark cycle of 12 h/24 h with free access to normal food and tap water. These animals were handled in accordance with the European Union directives for the animals protection (EEC Council 86/609) (Smith et al. 2007).

### 2.2 Plant Material and Extraction

The plant material used in our study consisted of the barks of the trunk of *Erythrophleum africanum*. Bark samples were harvested in February 2024 in the locality of Pitoa, Department of Benue (Northern Region, Cameroon). The identification of the plant sample was made by Doctor FAWA Guidawa, Botanist of the Department of Biological Sciences of the Faculty of Sciences of the University of Ngaoundere. The collected barks were cut into small pieces, then dried in the shade at room temperature for six (06) weeks.

The process followed to obtain the powder fractions was that of controlled differential sieving (Deli et al. 2019). The dried bark samples were pounded using a mortar to reduce their size and facilitate their passage into an electric grinder. The powder obtained was divided into two equal halves, one of which was kept and the other was used for particle size separation. Thus, 200 g of powder were gently poured into the upper sieve of a “BIOBASE” type sieve and sieving was carried out in permanent vibratory mode for 10 min. The powder fraction retained on each sieve was collected and then weighed to determine the mass fraction of each particle size class. Thus, we obtained from this sieving process, five (05) particle size fractions of powders (> 500 μm; 500–300 μm; 300–120 μm; 120–50 μm and ≤ 50 μm). The particle size fractions and the unsieved powder were finally packed in polyethylene bags and stored at 10°C until experimentation.

### 2.3 Induction and Treatment of Type 2 Diabetes

Induction was done using a high‐calorie sugar diet (HSD) (Table 1), according to the MACAPOS 1 (Maize, Cassava, palm Oil and Sucrose) model (Kamgang et al. 2005) modified by the additional intraperitoneal administration of dexamethasone (NDC 57319–519‐05, Phoenix) at 0.2 mg/kg bw (Niu et al. 2018), once a week from the third week to the end. The treatment itself was based on the administration of metformin, particle size fractions (≤ 50 μm, 120–50 μm) and unsieved powder of *Erythrophleum africanum* trunk bark.

**TABLE 1:** Composition of different diets (Kamgang et al. 2005).

Ingredients	Normal Diet (g/kg)	HSD (g/kg)
Corn	250	330
Wheat	400	260
Soya	150	100
Fish powder	100	30
Sucrose	—	200
Palm oil	—	60
Bone powder	10	10
Palm kernel meals	50	—
Cassava	—	100
Salt	05	05
Vitamin mix	05	05
Energy kcal/kg	3400	4340

Abbreviation: HSD, high‐caloric‐sucrose Diet.

Thirty (30) male rats were divided into six (06) groups of five (05) rats each and treated daily for 7 weeks as follows:

- Group 1: normal control (NC) rats received a normal diet and distilled water (10 mL/kg/day);
- Group 2: diabetic control (DC) rats received a high‐caloric diet, 10 mL/kg/day of distilled water and sucrose (4 g/kg bw);
- Group 3: Metformin‐treated (MET) rats received a high‐caloric diet, Metformin Hydrochloride (NDC 0378–6001‐91, Mylan Pharmaceuticals Inc.) (20 mg/kg bw) and sucrose (4 g/kg bw);
- Group 4: fractions‐treated (EAPF≤ 50 μm) rats received a high‐caloric diet, 300 mg/kg of the fraction (≤ 50 μm) of *E. africanum* trunk bark *per os* and sucrose (4 g/kg bw) *per os*;
- Group 5: fractions‐treated (EAPF120–50 μm) rats received a high‐caloric diet, 300 mg/kg of the fraction (120–50 μm) of *E. africanum* trunk bark *per os* and sucrose (4 g/kg bw);
- Group 6: Unsieved powder‐treated rats received a high‐caloric sucrose diet, 300 mg/kg of unsieved powdered *E. africanum* trunk bark *per os*, and 4 g/kg sucrose (Kamgang et al. 2008).

Dexamethasone (0.2 mg/kg bw) was administered intraperitoneally from the third week until the end of the seventh week of treatment to all animals, except the normal control group, which received physiological saline (5 mL/kg) intraperitoneally as well.

An orally induced hyperglycaemia test was performed at the end of the seventh week. This test consisted of taking a fasting blood glucose level and then administering a solution of D‐glucose (4 mg/kg bw per day). Blood glucose levels were measured 30 min, 60 min, 90 min, and 120 min after D‐glucose administration in all 5 rats in each group (Ngakou Mukam et al. 2023). At the end of the treatment, the rats were previously fasted for 15 h and then anesthetized by intraperitoneal injection of a diazepam (10 mg/kg)/ketamine (50 mg/kg) mixture (Fokam Tagne et al. 2025). After anesthesia, the animals were sacrificed by incision of the jugular vein of the neck, and blood was collected in dry tubes for the measurement of parameters such as blood lipids, transaminases, and oxidative stress parameters. Organs such as the heart, liver, pancreas, and kidneys of each animal were collected instantly after blood collection, then rinsed in a lactated ringer (RL) solution, drained, and weighed to evaluate their relative mass compared to the animal's carcass according to the following formula:

(1) $$\text{Relative organ weight}\;\left(\%\right)=\frac{\text{Organ weight}\;}{\text{Animal}\;\text{weight}}\times 100$$

A buffer was used for liver homogenates (20%). Each homogenate was centrifuged at 3000 ***g*** for 15 min, and the collected supernatant was stored at −20°C for the evaluation of oxidative stress parameters.

### 2.4 Biochemical Analysis

For the lipid profile, triglyceride (TG), total cholesterol (TC), and HDL cholesterol concentrations were determined using Fortress Diagnostics kits (BXC0317A, UK) following the manufacturer's protocols. LDL‐cholesterol concentration was calculated from total cholesterol (TC), triglyceride (TG), and HDL cholesterol values following the formula (Friedewald et al. 1972):

(2) $$\left[\mathrm{LDL}-\text{cholesterol}\right]=\left[\mathrm{TC}\right]-\left[\mathrm{HDL}-\text{chol}\right]-\frac{\left[\mathrm{TG}\right]}{5}$$

The atherogenic index (AI) was calculated following the formula (Castelli et al. 1983):

(3) $$\mathrm{AI}=\frac{\left[\mathrm{TC}\right]}{\left[\mathrm{HDL}-\text{chol}\right]}$$

Alanine Aminotransferase (ALT) and Aspartate Aminotransferase (AST) activities were determined using Fortress Diagnostics Kits (BXC0212A and BXC0202A, respectively) following the manufacturer's protocols.

Serum creatinine was determined using the kinetic method (Bartels and Cikes 1969). 100 μL of creatinine standard, 100 μL of distilled water, and 100 μL of sample were added to the standard tube, the blank tube, and the test tube, each containing 500 μL of the reaction medium. The mixture was homogenized, and the absorbances of the standard and test tubes were read at 500 nm against the blank at 30 and 90 s. The creatinine concentration was calculated as follows:

(4) $$\left[\text{Crea}\right]=\frac{A.\text{Sample}}{A.\mathrm{Std}}*\text{Conc}.\mathrm{Std}$$

where [Crea], creatinine concentration (mg/dL); A.Sample, absorbance of the sample; A.Std, absorbance of the standard; Conc.Std, standard concentration (200 mg/dL).

For malondialdehyde (MDA) Assay, 125 μL of trichloroacetic acid (TCA, 20%) and 250 μL of thiobarbituric acid (TBA, 0.67%) were added to test tubes containing 250 μL of Tris–HCl buffer (50 mM, pH 7.4) or homogenate. The tubes were capped with glass beads, heated to 90°C in a water bath for 10 min, then cooled in tap water and centrifuged at 3000 ***g*** at room temperature for 15 min. The supernatant was pipetted and the absorbance was read at 530 nm against the blank (Fokam Tagne et al. 2021). The MDA concentration in each sample was determined using the following formula:

(5) $$\left[\mathrm{MDA}\right]=\frac{\mathrm{\Delta DO}}{ɛ*L*m}$$

where [MDA], MDA concentration (mol/g of organs); ΔDO, optical density of the assay—optical density of the blank; L, optical path length (1 cm); ɛ, molar extinction coefficient (13,600 mol^−1^. cm^−1^); m, organ mass (g).

For nitric oxide (NO) determination, 400 μL of distilled water and 500 μL of Griess reagent were added to tubes containing 100 μL of samples or the blank. The mixture was homogenized and incubated in the dark for 10 min at room temperature. Absorbance was read using a spectrophotometer at 560 nm (Fermor et al. 2001). The nitrite concentration in each sample was determined using the following formula:

(6) $$\left[\text{Nitrite}\right]=\frac{\Delta \mathrm{DO}}{a*m}$$

where [Nitrite], nitrite concentration (μmol/g of organs); ΔDO, test OD—blank OD; a, slope of the calibration curve; m, organ mass.

For superoxide dismutase (SOD) Assay, 1666 μL of carbonate buffer (0.05 M, pH 10.2) and 200 μL of adrenaline (0.3 mM) were added to the test tubes containing 134 μL of sample. In the blank tubes, 1800 μL of carbonate buffer (0.05 M, pH 10.2) was added to 200 μL of adrenaline (0.3 mM). After homogenization of the different tubes, the absorbances of the test tubes were read against the blank at 480 nm at 20 and 80 s (Fokam Tagne et al. 2021). SOD activity was determined as follows:

(7) $$\%\text{inhibition}=100-\frac{\left(A20s-A80s\right)\text{test}}{\left(A20s-A80s\right)\text{blank}}*100$$

where A20s, Absorbance measured at 20 s; A80s, Absorbance measured at 80 s.

For catalase (CAT) Assay, 187.5 μL of phosphate buffer (0.1 mM; pH 7.5) was added to 12.5 μL of homogenates for the test tubes and to 12.5 μL of distilled water for the blank tube. 50 μL of hydrogen peroxide (50 mM) was added to each tube and incubated for 1 min at room temperature. 500 μL of potassium dichromate/glacial acetic acid (5%) was then added to all tubes and the mixture was heated at 100°C for 10 min. After cooling the individual tubes, the absorbance was read at 570 nm against the blank (Fokam Tagne et al. 2021). The specific activity of catalase was determined using the following formula:

(8) $$\mathrm{Act}\;\mathrm{CAT}=\frac{A.\text{test}-A.\text{blank}}{a*t*m}$$

where ActCAT, catalase activity (mM H_2_O_2_/min/g organs); A.test, absorbance of the test tubes; A.blank, absorbance of the blank tube; a, slope of the calibration curve; t, reaction time (1 min); m, organ mass (g).

For reduced glutathione Assay, 1500 μL of Ellman's reagent was added to 100 μL of homogenates for the test tubes or to 100 μL of Tris–HCl buffer (50 mM; pH = 7.4) for the blank tube. The mixture was homogenized and incubated at room temperature for 60 min. Absorbances were read against the blank at 412 nm (Fokam Tagne et al. 2021). GSH concentrations were determined according to the following formula:

(9) $$\left[\mathrm{GSH}\right]=\frac{A.\text{test}-A.\text{blank}}{ɛ*l*m}$$

where [GSH], GSH concentration (mol/g of organs); A.test, absorbance of the test tubes; A.blank, absorbance of the blank tube; L, optical path length (1 cm); ε, molar extinction coefficient (15,600 mol^−1^. cm^−1^); m, organ mass (g).

### 2.5 Statistical Analysis

The results obtained were expressed as mean ± standard error of mean (SEM). Data processing was carried out using Microsoft Excel 2021 software and their exploration was done using IBM SPSS Statistics 27 software. The analysis of variance test “Two‐way ANOVA” followed by Turkey's multiple comparison test was carried out using GraphPad prism 10.1.0 software.

## 3 Results

### 3.1 Effect of *Erythrophleum africanum* Trunk Bark Powder Fractions on Fasting Glycemia in Diabetic Rats

**FIGURE 1:**
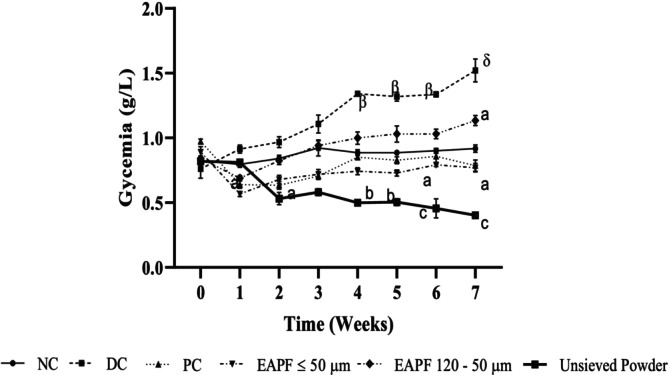
Glycemia of rats during 7 weeks of treatment. Values were expressed as mean ± SEM, (*n* = 5). Significant differences (^α^ *p* ≤ 0.05; ^β^ *p* ≤ 0.01; ^δ^ *p* ≤ 0.001) compared to normal control (NC) and (^a^ *p* ≤ 0.05; ^b^ *p* ≤ 0.01; ^c^ *p* ≤ 0.001) compared to diabetic control (DC). EAPF, Erythrophleum africanum powder fractions; PC, Positive control.

Fasting glycemia in the groups of animals at the start of the experiment (time T0) did not show any significant differences (with a mean value of approximately 0.85 ± 0.03 g/L (Figure 1)). Glycemia in diabetic rats increased significantly (*p* < 0.001) during the treatment compared with the normal control group. Blood glucose levels in the diabetic control group rose from 0.91 ± 0.03 g/L at the end of week 1 to 1.52 ± 0.08 g/L at the end of week 7. Fractions ≤ 50 μm, unsieved *E. africanum* powder and metformin (20 mg/kg) prevented the increase in blood glucose compared with diabetic control rats. Blood glucose values decreased from 0.56 ± 0.01 g/L, 0.81 ± 0.02 g/L and 0.63 ± 0.02 g/L at week 1 to 0.77 ± 0.03 g/L, 0.30 ± 0.10 g/L and 0.59 ± 0.19 g/L at week 7 for the groups receiving fractions ≤ 50 μm, unsieved *E. africanum* powder and metformin (20 mg/kg), respectively. In addition, fractions ≤ 50 μm (300 mg/kg) produced the best long‐term glycaemic control compared with unsieved powder (300 mg/kg) and metformin (20 mg/kg).

### 3.2 Effects of *Erythrophleum africanum* Trunk Bark Powder Fractions on Oral Glucose Tolerance

**FIGURE 2:**
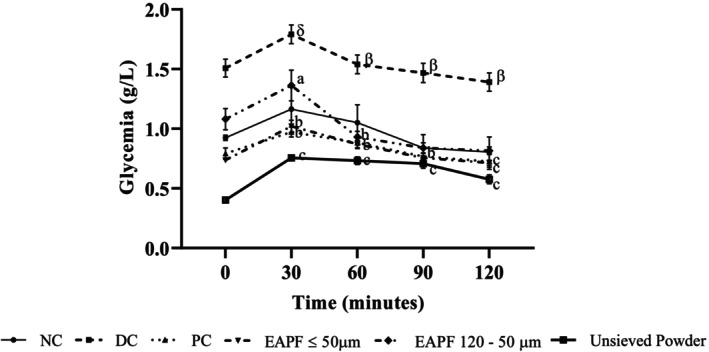
Glycemia variation in rats during the oral glucose tolerance. Values were expressed as mean ± SEM, (*n* = 5). Significant differences (^α^ *p* ≤ 0.05; ^β^ *p* ≤ 0.01; ^δ^ *p* ≤ 0.001) compared to normal control (NC) and (^a^ *p* ≤ 0.05; ^b^ *p* ≤ 0.01; ^c^ *p* ≤ 0.001) compared to diabetic control (DC). EAPF, Erythrophleum africanum powder fractions; PC, Positive control.

30 min after glucose administration (4 g/kg), the blood glucose values were as follows: 1.16 ± 0.2; 1.79 ± 0.07; 0.97 ± 0.04; 1.02 ± 0.05; 1.36 ± 0.12; 0.75 ± 0.02 g/L, respectively in the normal control, diabetic control, positive control, and test groups receiving *Erythrophleum africanum* powder fractions ≤ 50 μm, 50–120 μm and unsieved powder (300 mg/kg bw). These values were significantly decreased (*p* < 0.001) in animals treated with *E. africanum* powder fractions ≤ 50 μm, 50–120 μm, unsieved powder, or metformin and were 0.71 ± 0.02; 0.81 ± 0.03; 0.57 ± 0.03; 0.72 ± 0.06 g/L, respectively (Figure 2).

### 3.3 Effects of *Erythrophleum africanum* Trunk Bark Fractions Powder on Food Intake

**FIGURE 3:**
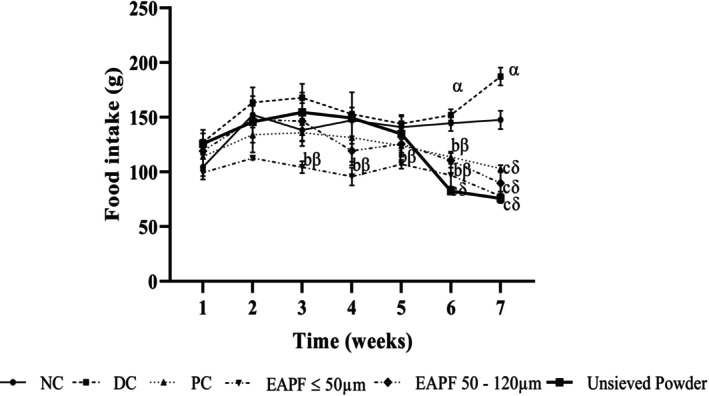
Food intake of rats during treatment. Values were expressed as mean ± SEM, (*n* = 5). Significant differences (^α^ *p* ≤ 0.05; ^β^p ≤ 0.01; ^δ^ *p* ≤ 0.001) compared to normal control (NC) and (^a^ *p* ≤ 0.05; ^b^ *p* ≤ 0.01; ^c^ *p* ≤ 0.001) compared to diabetic control (DC). EAPF, Erythrophleum africanum powder fractions; PC, Positive control.

During the first 4 weeks of treatment, food consumption of animals receiving the normal diet (Normal Control) showed no significant difference compared with that of animals receiving the high‐calorie diet (Diabetic Control). At the end of week 7, food consumption of animals receiving the high‐calorie diet and treated with distilled water (Diabetic Control) (187.25 ± 8.08 g) increased significantly (*p* < 0.05) compared with that of animals receiving the normal diet and distilled water (Normal Control) (147.50 ± 8.52 g) (Figure 3). However, in animals receiving the high‐calorie diet and treated with fractions (≤ 50 μm, 50–120 μm), unsieved powder, or metformin (20 mg/kg), food intakes were significantly reduced (*p* < 0.001) compared with the diabetic control. These values at the seventh week were 77.75 ± 4.25 g, 89.75 ± 10.91 g, 75.66 ± 4.05 g, and 103 ± 3.21 g in animals treated with powder fractions ≤ 50 μm, 50–120 μm, unsieved powder, and metformin, respectively.

### 3.4 Effects of Trunk Bark Powder Fractions of *Erythrophleum africanum* on Body Weight

**FIGURE 4:**
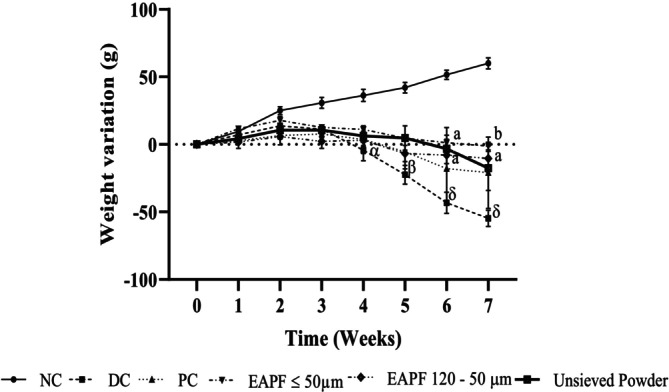
Variation of body weight of rats during the 7 weeks of treatment. Values were expressed as mean ± SEM, (*n* = 5). Significant differences: ^α^ *p* ≤ 0.05; ^β^ *p* ≤ 0.01; ^δ^ *p* ≤ 0.001) compared to normal control (NC) and ^a^ *p* ≤ 0.05; ^b^ *p* ≤ 0.01; ^c^ *p* ≤ 0.001 compared to diabetic control (DC). EAPF, Erythrophleum africanum powder fractions; PC, Positive control.

The weight of animals in the diabetic control, positive control, and animals receiving fractions ≤ 50 μm, 120–50 μm, unsieved powder decreased significantly (*p* < 0.001) compared to the normal control (Figure 4).

### 3.5 Effects of Trunk Bark Powder Fractions of *Erythrophleum africanum* on Relative Mass of Organs

The effect of the concomitant administration of the sugary high‐calorie diet and the powder fractions of the bark of the trunk of *Erythrophleum africanum* on the relative mass of the organs, namely the heart, liver, pancreas, and kidneys, is recorded in Table 2 below. It is evident from this table that no significant variation was induced in the different groups of animals.

**TABLE 2:** Relative mass of the heart, liver, pancreas, and kidneys.

Groups	Relative mass (%)			
	Heart	Liver	Pancreas	Kidneys
NC	0.38 ± 0.03	3.38 ± 0.14	0.38 ± 0.14	0.75 ± 0.03
DC	0.47 ± 0.05^α^	4.61 ± 0.10^β^	0.50 ± 0.10	0.81 ± 0.00^α^
MC	0.44 ± 0.01^α^	4.49 ± 0.35^β^	0.36 ± 0.06	0.78 ± 0.06
EAPF ≤ 50 μm	0.37 ± 0.04**	4.02 ± 0.07*	0.37 ± 0.05	0.71 ± 0.02**
EAPF 120–50 μm	0.36 ± 0.03**	4.35 ± 0.25^β^	0.41 ± 0.04	0.69 ± 0.02^α^**
Unsieved powder	0.35 ± 0.02**	4.11 ± 0.14^β^	0.41 ± 0.09	0.64 ± 0.03^β^**

*Note:* Values were expressed as mean ± SEM, (*n* = 5). Significant differences (^α^ *p* < 0.05; ^β^ *p* < 0.01) compared to normal control (NC) and (*p < 0.05; **p < 0.01) compared to diabetic control (DC).

Abbreviations: EAPF, Erythrophleum africanum powder fractions; PC, Positive control.

### 3.6 Effects of Trunk Bark Powder Fractions of *Erythrophleum africanum* on the Lipid Profile of Diabetic Rats

At the end of treatment, triglycerides, total cholesterol, LDL‐cholesterol, HDL‐cholesterol, and atherogenic index were 52.36 ± 7.00, 141.4 ± 9.72, 38.22 ± 11.18, 92.7 ± 2.38, and 1.53 ± 0.14, respectively, in the normal control. In animals fed the high‐calorie diet and treated with distilled water, these values were 125.66 ± 3.60, 260.42 ± 16.71, 171.60 ± 16.84, 62.91 ± 1.16, and 4.15 ± 0.34, respectively, for triglycerides, total cholesterol, LDL‐cholesterol, HDL‐cholesterol, and atherogenic index. These values were significantly decreased (*p* < 0.001) in animals treated with *E. africanum* powder fractions and metformin compared to diabetic controls (Table 3).

**TABLE 3:** Serum levels of triglycerides (TG), total cholesterol (TC), HDL‐cholesterol (HDLc), LDL‐cholesterol (LDLc), and atherogenic index (AI) in rats at the end of treatment.

Groups	TG	TC	HDLc	LDLc	AI
NC	52.36 ± 7.00	141.4 ± 9.72	92.7 ± 2.38	38.22 ± 11.18	1.53 ± 0.14
DC	125.66 ± 3.60^β^	260.42 ± 16.71^β^	62.91 ± 1.16^α^	171.60 ± 16.84^β^	4.15 ± 0.34^β^
MC	74.8 ± 10.77**	157.2 ± 6.77**	108.46 ± 4.69**	33.77 ± 10.15**	1.45 ± 0.11**
EAPF ≤ 50 μm	57.83 ± 10.54**	149.76 ± 19.61**	120.63 ± 1.69**	7.86 ± 1.34**	1.24 ± 0.17**
EAPF 50–120 μm	74 ± 6.72**	149.93 ± 8.45**	109.23 ± 2.74**	25.9 ± 10.59**	1.37 ± 0.10**
Unsieved powder	55.43 ± 12.86**	155.46 ± 16.33**	119.7 ± 3.36**	24.68 ± 15.57**	1.30 ± 0.13**

*Note:* Values were expressed as mean ± SEM, (*n* = 5). Significant differences (^α^ *p* < 0.05; ^β^ *p* < 0.01) compared to normal control (NC) and (**p < 0.01) compared to diabetic control (DC).

Abbreviations: EAPF, Erythrophleum africanum powder fractions; MC, metformin control.

### 3.7 Effects of Trunk Bark Powder Fractions of *Erythrophleum africanum* on Hepatic and Renal Parameters in Diabetic Rats

A significant increase (*p* < 0.05) of serum ALAT and ASAT activities and creatininemia was observed in rats in the diabetic control group compared with normal control (Table 4). However, administration of fractions ≤ 50 μm, 50–120 μm, unsieved powder, and metformin (20 mg/kg) resulted in a significant (*p* < 0.05) reduction in these parameters in rats in these respective groups compared with rats in the diabetic control group.

**TABLE 4:** Serum transaminase and creatinine levels in rats after treatment.

Groups	ALAT (U/L)	ASAT (U/L)	Creatinine (mg/dL)
NC	37.55 ± 2.48	68.11 ± 1.70	0.66 ± 0.04
DC	55.46 ± 3.69^α^	90.05 ± 8.83^α^	1.15 ± 0.11^β^
MC	42.90 ± 4.09*	72.83 ± 6.52*	0.76 ± 0.07*
EAPF ≤ 50 μm	38.38 ± 7.09**	70.91 ± 1.82*	0.73 ± 0.09*
EAPF 120–50 μm	45.63 ± 1.15*	78.93 ± 3.35*	0.87 ± 0.06*
Unsieved powder	45.21 ± 2.28*	86.7 ± 5.33	1.03 ± 0.25^α^

*Note:* Values were expressed as mean ± SEM, (*n* = 5). Significant differences (^α^ *p* < 0.05; ^β^ *p* < 0.01) compared to normal control (NC) and (*p < 0.05; **p < 0.01) compared to diabetic control (DC).

Abbreviations: DC, Diabetes control; EAPF, Erythrophleum africanum powder fractions.

### 3.8 Effects of Trunk Bark Powder Fractions of *Erythrophleum africanum* on Oxidative Stress Parameters

Serum malondialdehyde (MDA) levels were 46.68 ± 0.26, 113.86 ± 2.28, 37.47 ± 0.38, 16.85 ± 0.53, 18.37 ± 0.42, and 59.22 ± 1.55 μmol/L in the normal control, diabetic control, positive control, and animals treated with *E. africanum* powder fractions or unsieved powder, respectively. In the liver, these values were, respectively, 144.02 ± 9.17, 201.52 ± 5.34, 130.62 ± 7.86, 113.04 ± 8.18, 131.95 ± 12.38, and 132.94 ± 13.76 μmol/g of liver in the normal control, the diabetic control, the positive control, and the animals treated with *E. africanum* powder fractions or with the unsieved powder (Table 5).

**TABLE 5:** Serum and liver of oxidative stress parameter levels in rats after treatment.

	MDA (μmol/L)	NO (nmol/L)	CAT (mM/min/L)	SOD (Unit/L)	GSH (μmol/L)
Serum					
Normal control	46.68 ± 0.26	215.87 ± 14.68	107.88 ± 12.37	363.16 ± 6.07	59.58 ± 2.12
Diabetic control	113.86 ± 2.28^β^	58.77 ± 8.30^β^	52.64 ± 2.85^β^	122.81 ± 14.03^β^	11.95 ± 0.40^α^
Positive Control	37.47 ± 0.38*	201.68 ± 16.72**	90.74 ± 11.53^β^*	374.15 ± 3.03**	54.48 ± 2.00*
*EAPF* ≤ 50 μm	16.85 ± 0.53^β^**	204.52 ± 14.64**	131.21 ± 15.84^β^	484.21 ± 10.95**	72.20 ± 4.38**
*EAPF* 50–120 μm	18.37 ± 0.42^β^*	134.48 ± 4.95**	95.74 ± 12.26*	409.65 ± 3.50**	55.34 ± 1.82*
Unsieved powder	59.22 ± 1.55^α^**	137.32 ± 6.13**	79.55 ± 10.13*	357.89 ± 6.07**	62.64 ± 2.39**

	MDA (μmol/g)	NO (nmol/g)	CAT (mM/min/g)	SOD (U/g)	GSH (μmol/g)
Liver					
Normal Control	144.02 ± 9.17	599.08 ± 19.59	203.69 ± 10.71	701.75 ± 35.08	127.44 ± 8.08
Diabetic Control	201.52 ± 5.34^β^	265.47 ± 7.26^β^	97.74 ± 8.59^β^	508.77 ± 36.47^β^	67.76 ± 9.09^β^
Positive Control	130.62 ± 7.86**	383.77 ± 18.46^β^*	171.55 ± 7.13**	763.16 ± 28.94**	116.17 ± 7.96**
*EAPF* ≤ 50 μm	113.04 ± 8.18**	613.57 ± 14.00**	197.74 ± 14.41**	859.65 ± 23.20**	128.05 ± 11.49**
*EAPF* 50–120 μm	131.95 ± 12.38**	397.97 ± 14.48^β^*	187.02 ± 10.49**	824.56 ± 21.20**	136.14 ± 4.25**
Unsieved powder	132.94 ± 13.76**	286.76 ± 6.78^β^	135.83 ± 12.75*	850.88 ± 17.54**	93.74 ± 8.22*

*Note:* Values were expressed as mean ± SEM, (*n* = 5). Significant differences (^α^ *p* < 0.05; ^β^ *p* < 0.01) compared to normal control and (*p < 0.05; **p < 0.01) compared to diabetic control.

Abbreviations: CAT, catalase; EAPF, Erythrophleum africanum powder fractions; GSH, reduced glutathione; MDA, malondialdehyde; NO, nitric oxide; SOD, superoxide dismutase.

Serum catalase activities were 107.88 ± 12.37, 52.64 ± 2.85, 90.74 ± 11.53, 131.21 ± 15.84, 95.74 ± 12.26, and 79.55 ± 10.13 μmol/L in the normal control, diabetic control, positive control, and animals treated with *E. africanum* powder fractions or unsieved powder, respectively. In the liver, these values were, respectively, 203.69 ± 10.71, 97.74 ± 8.59, 171.55 ± 7.13, 197.74 ± 14.41, 187.02 ± 10.49, and 135.83 ± 12.75 μmol/g of liver in the normal control, the diabetic control, the positive control, and the animals treated with *E. africanum* powder fractions or with the unsieved powder (Table 5).

The serum SOD activities were 363.16 ± 6.07, 122.81 ± 14.03, 374.15 ± 3.03, 484.21 ± 10.95, 409.65 ± 3.50, and 357.89 ± 6.07 μmol/L in normal control, diabetic control, positive control, and animals treated with *E. africanum* powder fractions or unsieved powder, respectively. In the liver, the SOD activities were 701.75 ± 35.08, 508.77 ± 36.94, 763.16 ± 28.94, 859.65 ± 23.20, 824.56 ± 21.20, and 850.88 ± 17.54 μmol/g liver in the normal control, diabetic control, positive control, and animals treated with *E. africanum* powder fractions or unsieved powder, respectively (Table 5).

The levels of reduced glutathione in the liver were 127.44 ± 8.08, 67.76 ± 9.09, 116.17 ± 7.96, 128.05 ± 11.49, 136.14 ± 4.25, and 93.74 ± 8.22 μmol/g liver in normal control, diabetic control, positive control, and animals treated with *E. africanum* powder fractions or unsieved powder, respectively. In serum, GSH values were 59.58 ± 2.12, 11.95 ± 0.40, 54.48 ± 2.00, 72.20 ± 4.38, 55.34 ± 1.82, and 62.64 ± 2.39 μmol/L in normal control, diabetic control, positive control, and animals treated with *E. africanum* powder fractions or unsieved powder, respectively (Table 5).

Serum nitric oxide levels were 215.87 ± 14.68, 58.77 ± 8.30, 201.68 ± 16.72, 204.52 ± 14.64, 134.48 ± 4.95, and 137.32 ± 6.13 μmol/L in normal control, diabetic control, positive control, and animals treated with *E. africanum* powder fractions or unsieved powder, respectively. In the liver, the NO values were 599.08 ± 19.59, 265.47 ± 7.26, 383.77 ± 18.46, 613.57 ± 14.00, 397.97 ± 14.48, and 286.76 ± 6.78 μmol/g liver in the normal control, diabetic control, positive control, and animals treated with *E. africanum* powder fractions or unsieved powder, respectively (Table 5).

## 4 Discussion

Due to the epidemiological, etiological, and risk factor aspects of type 2 diabetes in humans, it is essential to choose an appropriate animal model in order to reproduce the human physiological state in order to test antidiabetic phytomedicines (Vialettes 2021). One of the features of this model would be the possibility of the appearance of insulin resistance. Several studies have shown that when rats are subjected to a sugary high‐calorie diet, they easily develop insulin resistance (Kamgang et al. 2005). Most commonly, dexamethasone (exogenous glucocorticoid) is combined with this diet because of its ability to promote the development of insulin resistance by reducing the number and activity of glucose transporters (Bastin and Andreelli 2020; Ngounou et al. 2021). Thus, the animal model of type 2 diabetes induced by a hypercaloric‐sucrose diet combined with the administration of dexamethasone (0.2 mg/kg) is the one that would reproduce the metabolic characteristics of type 2 diabetes in humans. *Erythrophleum africanum* is a medicinal plant used in the treatment of several pathologies, including diabetes. The present study aimed to evaluate the potential antidiabetic and antioxidant effect of *Erythrophleum africanum* trunk bark powder fractions on diet‐ and dexamethasone‐induced type 2 diabetes in rats.

Fasting blood glucose levels of animals after concomitant administration of the sugary high‐calorie diet and *Erythrophleum africanum* powder fractions decreased from the third to the seventh week. The substances contained in *E. africanum* are thought to be responsible for this pharmacological activity. Indeed, some compounds such as saponins, flavonoids, polyphenols, tannins, and cardiac glycosides have been highlighted by a phytochemical analysis of the aqueous extract of the stem bark of this plant (Tukur et al. 2022). Previous work has suggested the existence of a probable link between the phytochemical nature of the compounds present in plants and blood glucose levels (Bi et al. 2008). Substances such as polyphenols and flavonoids are generally recognized as being able to regulate blood glucose levels by increasing the expression of GLUT‐2 in pancreatic β‐cells and also increasing the expression of GLUT‐4 as well as promoting the inhibition of α‐glucosidase (Ansarullah et al. 2011; Hanhineva et al. 2010; Soares et al. 2017). Saponins are recognized for their role in reducing blood sugar by inhibiting intestinal absorption of glucose, preventing glucose storage, which would then cause insulin secretion (Kambouche et al. 2009). The efficacy of our plant is more marked than the efficacy of metformin and suggests that in addition to the pathway of action of metformin, the plant may have other pathways of action. Indeed, metformin acts on the liver by reducing hepatic glucose production via the inhibition of gluconeogenesis (Cladera et al. 2024; Vezza et al. 2023). It also contributes to improving the sensitivity of insulin receptors (Foretz et al. 2021; Koffert et al. 2017).

The loss of body weight observed during the experiment in animals in the negative control group could be explained by the hydrolysis of protein and lipid reserves at the tissue level due to the inability of cells to use blood glucose (Sidibeh 2018). Powder fractions of the bark of the trunk of *Erythrophleum africanum* also caused a decrease in weight. This effect is related to the decrease in food intake, which could be explained by the influence of the extract on the appetite of rats (Jouad et al. 2003; Kamgang et al. 2008). It is possible that these fractions contain polysaccharides which, once in the body, could be involved in reducing food consumption by replacing glucose, thus reducing cellular glucose requirements.

The vast majority of substances administered to the body orally (*per os*) are distributed to the liver to undergo biotransformation (presystemic metabolism) before entering systemic circulation and subsequently eliminated (Naud et al. 2015). The heart, being the central organ of the circulatory system of the organism of higher animals, can also be subjected to the toxic effect of substances. Analysis of the relative mass of the organs studied in the present study showed that they are all identical. This result suggests that the condition of the organs (heart, liver, pancreas, and kidneys) was not significantly modified by the treatment administered during the entire experiment.

In the diabetic control, we observed a dyslipidemia marked by high levels of triglycerides, total cholesterol, and LDL‐cholesterol and a low level of HDL‐cholesterol compared to the normal control, which would be associated with insulin resistance. The trunk bark fractions powder of *Erythrophleum africanum* improved this lipid profile by inducing an increase in the level of HDL‐cholesterol and a decrease in the level of triglycerides and LDL‐cholesterol. The inhibition of lipoprotein lipase activity prevents the hydrolysis of triglycerides into glycerol and fatty acids (Charrière 2021). The preventive action of *E. africanum* on the lipid profile would result from its capacity to inhibit lipoprotein lipase activity. The trunk bark fractions powder of *E. africanum* could act by reducing cholesterol biosynthesis by inhibiting the activity of 3‐hydroxy‐3‐methyl‐glutaryl coenzyme A reductase (HMG‐CoA reductase) (Pierrot and Octave 2014). The total cholesterol/HDL cholesterol ratio is an atherogenic index considered a very useful means to predict cardiovascular dysfunction (Castelli et al. 1983). An index less than or equal to 3 is synonymous with the absence of atherogenic risk, while an index greater than 4 in a subject represents a major risk of cardiovascular damage (Liu et al. 2024). The trunk bark fractions powder of *Erythrophleum africanum* showed an index well below 3, which would suggest a protective action of these extracts on the heart and its vascularization.

Since alanine aminotransferase (ALAT) and aspartate aminotransferase (ASAT) are enzymes of cytoplasmic origin, any cell necrosis, increased membrane permeability of hepatocytes, or destruction of the hepatic parenchyma can lead to an increase in their serum levels (Alkalah 2017; Jodynis‐Liebert et al. 2010). Creatinine remains a common semiological parameter for establishing the diagnosis of renal function (Dussol 2011). Thus, any substance that can modify the different renal functions would lead to a modification of the plasma concentration of creatinine (Filler et al. 2014; Gowda et al. 2010). The results show that the administration of the sugary high‐calorie diet coupled with dexamethasone induced a significant increase in ALAT, ASAT, and creatinine levels in animals in the diabetic control group. The powder fractions of *Erythrophleum africanum* did not cause an increase in the serum levels of these enzymes, which therefore suggests that the treatments are not toxic. Bioactive compounds such as flavonoids and saponins could be at the origin of these hepatoprotective and nephroprotective effects, as shown by El Khasmi and Farh (2022); Kouadio et al. (2022).

Diabetes mellitus is characterized by an overproduction of reactive oxygen species that is thought to arise from the autoxidation of glucose, thus leading to a significant level of oxidative stress (Boyer et al. 2014; Delattre et al. 2001). Malondialdehyde (MDA) is considered one of the most studied subtractive oxidation intermediates to assess the scope of cellular oxidation (Favier 2003; Lee et al. 2012). Thus, the significant increase in MDA concentration in the diabetic control would suggest the onset of oxidative stress due to the high production of free radicals induced by the administration of dexamethasone. Dexamethasone causes insulin resistance and hyperlipidemia leading to cellular lipotoxicity and glucotoxicity responsible for the overproduction of free radicals and therefore a weakening of antioxidant enzymes (CAT, SOD, GSH) in tissues (Ellah et al. 2017; Keeney et al. 1993). The low concentration of NO in these animals of the diabetic control group would result on the one hand from the effects of dexamethasone which, by causing insulin resistance, would have inhibited the activation of NOS (the NO synthesis enzyme), on the other hand from a pronounced use of NO as an antioxidant (Fofié et al. 2018). The increase in CAT, SOD, GSH activity, NO level and the decrease in MDA level observed in the serum and liver homogenate of rats treated with *Erythrophleum africanum* powder fractions suggest that the latter would have an anti‐radical activity and that they would improve either insulin sensitivity, or NOS activity (by stimulating its synthesis or inactivating its inhibitor), or by ensuring the availability of L‐arginine (NO precursor) (Fofié et al. 2018; Tchamadeu et al. 2022). Some studies link antioxidant activity to the phytochemical composition of plant species (Rivera‐Yañez et al. 2018; Yadav et al. 2015). In fact, bioactive compounds such as flavonoids, polyphenols, saponins, and tannins contained in *E. africanum* act by trapping free radicals and stimulating the biosynthesis of antioxidants, hence its antiradical power (Olalekan 2015; Tukur et al. 2022).

## 5 Conclusion

The aim of this work was to evaluate the antidiabetic and antioxidant effects of trunk bark powder fractions of *Erythrophleum africanum* on type 2 diabetes induced by a high‐calorie diet and dexamethasone in rats. This plant showed antihyperglycemic properties in hyperglycemic rats, hypolipidemic activity, and antioxidant activity. This study attests that *E. africanum* has properties against type 2 diabetes mellitus and provides arguments in favor of its use in traditional medicine in the treatment of diabetes. In order to better elucidate the action mechanisms of powder fractions of the *E. africanum* trunk bark in the treatment of diabetes mellitus, we should in the future assess the action of the different compounds of our powder fractions on β cells of the pancreatic islets of Langerhans, on liver neoglucogenesis, and enterocytes, as well as the potential toxic effects of this plant. Once these mechanisms are elucidated, *E. africanum* powder could be used as food supplements in diabetic patients.

## Author Contributions

**Mathieu Sini:** investigation (equal), methodology (equal), writing – original draft (equal). **Faustin Dongmo:** conceptualization (equal), formal analysis (equal), investigation (equal), methodology (equal), writing – review and editing (equal). **Clemence Mvongo:** formal analysis (equal), methodology (equal), writing – original draft (equal), writing – review and editing (equal). **Pierre Jidibe:** investigation (equal), methodology (equal), resources (equal). **Angèle Foyet Fondjo:** formal analysis (equal), investigation (equal), resources (equal), writing – review and editing (equal). **Paul Aimé Noubissi:** conceptualization (equal), formal analysis (equal), methodology (equal), writing – review and editing (equal). **René Kamgang:** conceptualization (equal), project administration (equal), supervision (equal), validation (equal). **Michel Archange Fokam Tagne:** conceptualization (equal), formal analysis (equal), investigation (equal), project administration (equal), methodology (equal), writing – review and editing (equal), Validation (equal).

## Conflicts of Interest

The authors declare no conflicts of interest.

## Data Availability

The data that support the findings of this study are available from the corresponding author upon reasonable request.
